# Socio-Demographic and Dietary Factors Associated with Excess Body Weight and Abdominal Obesity among Resettled Bhutanese Refugee Women in Northeast Ohio, United States

**DOI:** 10.3390/ijerph110706639

**Published:** 2014-06-25

**Authors:** Madhav P. Bhatta, Lori Assad, Sunita Shakya

**Affiliations:** Department of Biostatistics, Environmental Health Sciences, and Epidemiology, College of Public Health, Kent State University, Kent, OH 44242, USA; E-Mails: lassad@kent.edu (L.A.); sshakya@kent.edu (S.S.)

**Keywords:** Bhutanese refugees, U.S., excess body weight, abdominal obesity, socio-demographic, dietary, risk factors

## Abstract

Studies of obesity and related health conditions among the Bhutanese, one of the largest refugee groups resettled in the United States in the past five years, are limited. This study examined the factors associated with excess body weight (body mass index ≥ 23 kg/m^2^) and abdominal obesity (waist circumference > 80 cm) in a community-based sample of 18–65 year old Bhutanese refugee women in Northeast Ohio. A Nepali-language questionnaire was used to measure socio-demographic and dietary factors. Height, weight, and waist circumference were measured to define excess body weight and abdominal obesity. The mean (±standard deviation) age of the 108 participants was 36.5 (±12.2) years and length of time in the U.S. was 19.4 (±11.9) months. Overall, 64.8% and 69.4% of the women had excess body weight and abdominal obesity, respectively. Age was significantly associated with both excess body weight (odds ratio: 1.10; 95% confidence interval: 1.05–1.16) and abdominal obesity (1.09; 1.04–1.14). Consuming meat (4.01; 1.14–14.60) was significantly associated with excess body weight but not abdominal obesity. These findings suggest the need for lifestyle and dietary change education programs among this new and vulnerable group to reduce the prevalence of excess body weight and abdominal obesity and their health consequences.

## 1. Introduction

The United States (U.S.) annually admits 50,000 to 70,000 individuals from protracted refugee situations for resettlement [1]. Refugees in protracted situations, defined as 25,000 or more refugees from the same country seeking asylum in another country for at least five consecutive years, “find themselves in a long-lasting and intractable state of limbo. Their lives may not be at risk, but their basic rights and essential economic, social, and psychological needs remain unfulfilled after years in exile” [2]. Third country resettlement is the only safe and viable solution for refugees in protracted situations when efforts for home country repatriation or host country integration have failed [3]. As part of its ongoing humanitarian commitment, the U.S. annually accepts the largest number of refugees for third country resettlement and the countries of origin of the resettled refugees vary from year to year.

The U.S. resettled refugees are an underserved, vulnerable, and health disparate group in part due to the circumstances of a protracted refugee situation, along with the issues related to third country resettlement [4]. Most studies of health issues in U.S. resettled refugee groups have primarily focused on infectious diseases, nutritional deficiencies, food insecurity, and mental health disorders [5,6,7,8,9]. However, limited studies of resettled refugees suggest obesity and related chronic diseases may be a significant public health issue among these groups [10,11,12]. Unlike infectious diseases, which are often diagnosed and treated during the pre- or post-arrival health screening of the resettled refugees, chronic conditions are not normally a part of the routine post-arrival screening protocol. Chronic conditions, however, require long term care and management, thus they represent a challenge in terms of continuity of care and management for the resettled refugees long after the initial resettlement support for resettled refugees ceases [13].

During the past five years, Bhutanese of Nepali origin were one of the largest groups of refugees resettled in the U.S. They accounted for 19% of the total 322,565 refugees admitted into the U.S. between 2008 and 2012 [14]. These resettled refugees were previously living in United Nations administered refugee camps in Eastern Nepal since the early 1990s when they fled their homes in Bhutan to escape political and ethnic persecution [15]. The resettled Bhutanese have joined the other 3.4 million ‘South Asian’ immigrants in the U.S. [16]. Among Asian ethnic groups in the U.S., South Asians have one of the highest rates of overweight/obesity [17], as defined by body mass index (BMI) ≥25 kg/m^2^. They also have a higher prevalence of abdominal (visceral) obesity, which is an independent risk factor for metabolic syndrome and related cardiometablic (diabetes and cardiovascular disease) risk, and greater insulin resistance at a lower BMI than other racial/ethnic groups in the U.S. [18,19,20,21]. As a result, South Asians have a higher prevalence of type 2 diabetes and cardiovascular disease at similar BMIs than other ethnic/racial groups [22,23,24,25,26,27]. Bhutanese refugees are likely to share a similar risk profile for obesity, metabolic syndrome, and cardiometabolic disorders with other South Asian immigrants to the U.S., potentially making them a high-risk group for these conditions.

Although health statistics on resettled Bhutanese refugees are limited, the prevalence of overweight/obesity among adults may be substantial. A clinic based study of adults with a mean age of 40 years reported a 52% prevalence of overweight/obesity [28]. The key limitations of that study included a small sample size (n = 65) and possible selection bias due to the sample being derived from a single clinic. To the best of our knowledge, there are currently no studies of overweight/obesity with population-level data in this group.

Community observations among Bhutanese refugees in Northeast Ohio suggested that the problem of increasing body weight may be a concern, particularly among women. Overweight/obesity and abdominal obesity among women, especially in urban areas, is a rising public health problem has been reported in Nepali and other South Asian populations [29,30]. Generally, traditional Bhutanese-Nepali diet consists of high carbohydrates from white rice and potatoes and relatively low in fruit and vegetable intake [29,31]. Moreover, among the resettled Bhutanese refugees adoption of new dietary practices such as greater incorporation of meat in the diet and frequent consumption of sweetened beverages (e.g., soft drinks and sweetened fruit juices) has been observed; such dietary practices were not regularly a part of their diet in refugee camps in Nepal [31,32]. Furthermore, there has been an increase in the sedentary lifestyle post-resettlement, especially in women [32]. Adoption of new dietary practices to an already high carbohydrate diet and an increased sedentary lifestyle among resettled Bhutanese refugee women may have implications for rising body weight and its health impact. Understanding the current extent of the overweight/obesity problem in this group is important to assess the level of risk and to develop effective clinical and public health intervention programs in this growing, vulnerable, and health disparate population in the U.S. In this research, we examined factors associated with excess weight and abdominal obesity in a community-based sample of 18–65 year old Bhutanese refugee women living in Northeast Ohio.

## 2. Methods

### 2.1. Participant Recruitment Procedure

The participant recruitment procedure for the study has been described in detail previously [32]. Briefly, between June and November 2011, a community-based cross-sectional study was conducted in a convenience sample of 120 Bhutanese refugee women aged 18–65 year living in the Akron and Cleveland metropolitan areas in Northeast Ohio. The participants for the study were recruited from the community with the assistance of a female community liaison, who herself was a resettled refugee and spoke the Nepali language, the *lingua franca* of the Bhutanese refugees. The liaison was a well-connected member of the community and was able to contact the potential participants using the networks and connections within the community to recruit women for the study. The community liaison recruited 8–10 women per study visit conducted in the home of one of the study participants, which was efficient in reducing transportation issues for study participants.

Each study visit consisted of obtaining informed consent, administration of the survey, and anthropometric measurements. During group sessions, a research assistant read to the participants and explained the contents of both English and Nepali language consent forms to assure full comprehension of the research protocol prior to the participant consenting to the study. Copies of both English and Nepali consent forms were provided to the participants who were also given time to read and ask questions.

To ensure privacy and confidentiality, the processes of written informed consent, survey administration, and anthropometric measurements were conducted in a separate room. Prior to obtaining the consent the participants were again provided an opportunity to ask questions about the study. Those agreeing to participate signed the consent form or marked with an X, if unable to write. Each participant was provided a $10 store gift card as an incentive for volunteering her time in the study. The study protocol was approved by the Kent State University Institutional Review Board (Kent State University Human Subject Research Protocol #: IRB 311-235).

### 2.2. Survey Instrument

Socio-demographic characteristics, dietary practices, and physical exercise were assessed using an interviewer administered Nepali questionnaire developed for the study. The questionnaire, first prepared in English, was translated into Nepali by a research assistant with fluency in both English and Nepali. The translation was reviewed and edited for content accuracy by the Principal Investigator, who is a native Nepali speaker with fluency in English. The questionnaire consisted of 58 items on socio-demographics, nutrition, dietary knowledge, attitudes, practices, and health status related measures. Trained native Nepali speaking research assistants administered the questionnaire in person to the participants.

Variables examined in the presented analysis include socio-demographic characteristics: age in years (continuous); length of time in the U.S. in months (continuous); marital status (married/widowed/divorced *vs.* single); number of children (none *vs.* any); religion (Hinduism *vs.* other); education (no formal education vs. some formal education); ability to read English (yes/no); and currently employed (yes/no). Any physical exercise (yes/no) was assessed by asking if the participants engaged in any kind of physical activities (e.g., walking, running, and exercising). Those with an affirmative response were followed up with whether it was more than 20 minutes a day [33]. Dietary measures included whether they consumed various types of meats, dairy products, and sweetened beverages including soft drinks, fruit drinks, and flavored drinks. Due to limited study sample size, we were unable to meaningfully analyze the relationships between excess weight and abdominal obesity with specific types of meats, dairy products or sweetened beverages. Therefore, meat, dairy and sweetened beverage consumption was dichotomized as yes or no for the present analysis.

### 2.3. Anthropometric Measurements

Anthropometric measurements included: weight using a calibrated digital scale; height measured using a stadiometer; and waist circumference using a measuring tape at the mid-point between the lower ribs and the anterior superior iliac spine, the site of greatest circumference [34]. To ensure consistency, anthropometric measures were taken by a single trained research assistant.

Because of the increased risk of cardiometabolic disorders at a lower BMI for Asian populations than Caucasians, the World Health Organization (WHO) in 2002 recommended new BMI standards for Asians: 18.5 to <23.0 kg/m^2^ as normal weight, 23.0 to <27.5 kg/m^2^ as a moderate-risk for public health action, and ≥27.5 kg/m^2^ as high-risk for public health action [35]. Similarly, in 2009 the Ministry of Health in India adopted BMI ranges of 23.0 to <25.0 as overweight and ≥25 kg/m^2^ as obesity [36]. Based on these recommendations and other studies [23,24,26,37,38,39] of increased risk of cardiometabolic disorders in South Asians at a lower BMI, those with a BMI ≥23 kg/m^2^ were defined as having excess body weight in this study. The waist circumference, used to assess abdominal obesity, cut-off of >80 cm was used to categorize those with abdominal obesity at an increased risk of cardiometabolic disorders [40,41].

### 2.4. Statistical Analysis

Of the 120 women recruited to participate in the study, 12 were excluded due to current pregnancy. For the present analysis, variables for marital status, number of children, religion and education were dichotomized by collapsing the meaningfully similar categories. For example, married, widowed, and divorced women were more likely to be similar to each other than the single women, thus were combined into one category. Frequency distribution and proportions were reported for categorical variables. Mean (±standard deviation (SD)), median, and range were reported for continuous variables. Univariable and multivariable logistic regression analyses were performed to assess the magnitude of association between the outcome variables and the potential risk factors. The multivariable models included age, employment status, drinking sweetened beverages and consuming meat. Due to the limited sample size and the significant correlation of age with marital status, number of children, education, and the ability to read English, age and employment status were the two socio-demographic variables included in the adjusted model along with drinking sweetened beverages and consuming meat. The crude and adjusted odds ratios (OR) and corresponding 95% confidence intervals (CI) are reported. All statistical significance were assessed at α = 0.05 level. SAS^®^ 9.2 was used for the data analysis [42].

## 3. Results

### 3.1. Sample Characteristics

The mean (±sd) age of the 108 participants was 36.5 (±12.2) years and the mean (±sd) length of time in the U.S. was 19.4 (±11.9) (range: 0.65–42.6) months (Table 1). Eighty-one percent of the women were married, divorced or widowed. The median number of children the women had was 2 (range: 0–7) with 77.8% of the women having at least one child. The majority of the women (71.3%) reported practicing the Hindu religion. Fifty-four percent of the women reported having had no formal education, and 55.6% of the women reported not being able to read English. Twenty-one percent of the women were currently employed. Thirty-one percent of the women reported engaging in some sort of physical activity. In terms of dietary practices, 20.4% reported being a vegetarian, 89.8% reported consuming dairy products, and 66.8% reported consuming sweetened beverages.

**Table 1:** Socio-demographic characteristics and dietary practices among 18–65 year old Bhutanese refugee women in Northeast Ohio, United States, 2011 (n = 108).

Characteristics	Mean (standard deviation) or n (%)				
	All	Body Mass Index, kg/m^2^		Waist Circumference, cm	
		≥23.0	<23.0	>80	≤80
Age, years	36.5 (12.1)	40.3 (11.0)	29.6 (11.2)	39.5 (11.3)	29.7 (11.2)
					
Length of time in the United States, months	19.4 (11.9)	19.3 (11.8)	19.7 (12.2)	19.5 (12.0)	19.1 (11.8)
Marital status					
Married/widowed/divorced	87 (80.6)	66 (75.9)	21 (21.8)	70 (80.5)	17 (19.5)
Single	21 (19.4)	4 (19.1)	17 (80.9)	5 (23.8)	16 (76.2)
Religion					
Hindu	77 (71.3)	48 (62.3)	29 (37.7)	51 (66.2)	26 (33.8)
Other	31 (29.7)	22 (71.0)	9 (29.0)	24 (77.4)	7 (22.6)
Number of children					
0	24 (22.2)	5 (20.8)	19 (79.2)	6 (25.0)	18 (75.0)
≥1	84 (77.8)	65 (77.4)	19 (22.6)	69 (82.1)	15 (17.9)
Education					
None	58 (53.7)	48 (82.8)	10 (17.2)	48 (82.8)	10 (17.2)
At least some formal schooling	50 (46.3)	22 (44.0)	28 (56.0)	27 (54.0)	23 (46.0)
Able to read English					
No	60 (55.6)	50 (83.3)	10 (16.7)	50 (83.3)	10 (16.7)
Yes	48 (44.4)	28 (58.3)	20 (41.7)	25 (52.1)	23 (47.9)
Currently employed					
No	85 (78.7)	60 (70.6)	25 (29.4)	63 (74.1)	22 (25.9)
Yes	23 (21.3)	10 (43.5)	13 (56.5)	12 (52.2)	11 (47.8)
Engage in physical activities					
No	75 (69.4)	47 (62.7)	28 (37.3)	50 (66.7)	25 (33.3)
Yes	33 (30.6)	23 (69.7)	10 (30.3)	25 (75.8)	8 (24.2)
Consume meat					
No	22 (20.4)	11 (50.0)	11 (50.0)	13 (59.1)	9 (40.9)
Yes	86 (79.6)	59 (68.6)	27 (31.4)	63 (72.1)	24 (27.9)
Consume dairy products					
No	11 (10.2)	8 (72.7)	3 (27.3)	8 (72.7)	3 (27.3)
Yes	95 (89.8)	62 (63.9)	35 (36.1)	67 (69.1)	30 (30.9)
Consume sweetened beverages					
No	36 (33.3)	19 (52.8)	17 (47.2)	23 (63.9)	13 (36.1)
Yes	72 (66.8)	51 (70.8)	21 (29.2)	52 (72.2)	20 (27.8)

### 3.2. Overall Anthropometric Measures

The mean (±sd) height and weight of the study participants were 1.52 (±0.07) m and 58.2 (±11.2) kg, respectively. The mean (±sd) BMI was 25.2 (± 4.6) (range: 15.8–40.2) kg/m^2^. Six percent of the participants were underweight (BMI > 18.5 kg/m^2^), 28.7% were normal weight (18.5 to < 23.0 kg/m^2^), and 64.8% had excess weight (≥23.0 kg/m^2^). The mean (±sd) waist circumference was 85.9 (±4.7) (range: 57.2–113.0) cm. Sixty-nine percent of the women had a waist circumference >80 cm (abdominal obesity).

### 3.3. Excess Body Weight

In the univariable analyses, increasing age, being married/widowed/divorced, having one or more child, not having a formal education, not being able to read English, and not being currently employed were significantly associated with excess weight compared to women who were single, had no children, had some formal education, reported being able to speak English, and were currently employed (Table 2). Length of time in the U.S. was not associated with excess body weight in this study.

**Table 2:** Univariable logistic regression analyses of factors associated with excess weight (body mass index (BMI) ≥ 23.0 kg/m^2^) and abdominal obesity (waist circumference > 80 cm) among 18–65 year old Bhutanese refugee women in Northeast Ohio, United States, 2011 (n = 108).

Characteristic	Excess Body Weight (BMI ≥ 23.0 kg/m^2^) *				Abdominal Obesity (Waist Circumference > 80 cm) **		
	cOR ^§^	95 % CI ^§§^	*p*-value		cOR^§^	95 % CI ^§§^	*p*-value
Age per year increase	1.09	1.05–1.14	<0.0001	1.08		1.04–1.13	0.0003
Length of time in the United States per months increase	0.99	0.96–1.03	0.8615	1.00		0.97–1.04	0.8748
Marital status							
Married/Widowed/ Divorced *vs.* Single	13.36	4.04–44.12	<0.0001	13.2		4.23–41.01	<0.0001
Religion							
Hindu *vs.* Other	0.68	0.28–1.67	0.3970	0.57		0.22–1.50	0.2571
Number of children							
≥1 *vs.* 0	13.00	4.29–39.44	<0.0001	13.80		4.69–40.62	<0.0001
Education							
None *vs.* At least some formal schooling	6.12	2.53–14.74	<0.0001	4.09		1.70–9.85	0.0017
Able to read English							
No *vs.* Yes	7.00	2.88–17.03	<0.0001	4.60		1.90–11.14	0.0007
Currently employed							
No *vs.* Yes	3.12	1.21–8.04	0.0186	2.63		1.01–6.80	0.0468
Engage in physical activities							
No *vs.* Yes	0.73	0.30–1.76	0.4818	0.64		0.25–1.62	0.3469
Consume meat							
Yes *vs*. No	2.19	0.84–5.66	0.1075	1.79		0.67–4.73	0.2409
Consume dairy products							
Yes *vs.* No	0.66	0.17–2.67	0.5642	0.84		0.21–3.38	0.8033
Consume sweetened beverages							
Yes *vs.* No	2.17	0.95–4.98	0.0664	1.47		0.63–3.45	0.3767

* Reference group: BMI < 23.0 kg/m^2^; ** Waist Circumference ≤ 80 cm; ^§^ Crude Odds Ratio; ^§§^ 95% confidence interval.

In a multivariable logistic regression model that included age, employment status, meat consumption and drinking sweetened beverages, increasing age (OR = 1.10; 1.05–1.16) and consuming meat (OR = 4.01; 1.14–14.60) were significantly associated with excess body weight (Table 3).

**Table 3:** Multivariable logistic regression analyses of factors associated with excess weight (body mass Index (BMI) ≥ 23.0 kg/m^2^) and abdominal obesity (waist circumference > 80 cm) among 18–65 year old Bhutanese refugee women in Northeast Ohio, United States, 2011 (n = 108).

Factor	Excess Weight (BMI ≥ 23.0 kg/m^2^) *			Abdominal Obesity (Waist Circumference > 80 cm) **	
	aOR (95% CI) ^§^	*p*-value		aOR (95% CI) ^§^	*p*-value
Age per year increase	1.10 (1.05–1.16)	<0.0001		1.09 (1.04–1.14)	0.0003
Currently unemployed *vs.* employed	1.86 (0.64–5.37)	0.2547		1.65 (0.59–4.63)	0.2894
Consume sweetened beverages *vs.* no	2.04 (0.76–5.45)	0.1554		1.25 (0.47–3.29)	0.8827
Consume meat *vs.* no	4.01 (1.14–14.60)	0.0303		2.85 (0.87–9.30)	0.0835

* Reference group: BMI < 23.0 kg/m^2^; ** Waist Circumference ≤ 80 cm; ^§^ Adjusted Odds Ratio (95% confidence interval); adjusted for the variables listed above.

### 3.4. Abdominal Obesity

Similarly, in the univariable analyses increasing age, being married/widowed/divorced, having one or more child, having no formal education, not being able to read English, and not being currently employed were statistically significantly associated with abdominal obesity compared to women who were single, had no children, had some formal education, reported being able to speak English, and were currently employed (Table 2). In a multivariable model that included age, employment status, meat consumption and drinking sweetened beverages, only increasing age (OR = 1.09; 1.04–1.14) was significantly associated with abdominal obesity (Table 3).

## 4. Discussion and Conclusions

In one of the first community-based studies of risk factors for excess body weight and abdominal obesity among resettled Bhutanese refugee women in the U.S., we observed a significant proportion of women with excess total body weight (65%) and abdominal obesity (69%) which puts them at an increased risk of cardiometabolic disorders. We observed a statistically significant association between several socio-demographic characteristics and dietary factors with both excess body weight and abdominal obesity in this group of women. However, we did not observe an association between the length of time in the U.S. or physical activity with either measure of obesity in this study. These findings are noteworthy in that the issues of excess body weight and related chronic conditions among Bhutanese resettled refugees as potential health concerns have not garnered much research or practice attention in the U.S. or other western countries.

Although this group may have a lower prevalence of obesity compared to the general U.S. population when using the standard obesity criteria of BMI ≥30 kg/m^2^, it may not be an appropriate comparison due to the fact that South Asians have higher risk of cardiometabolic disorders at a lower BMI than other ethnic groups. Thus, the high prevalence of excess body weight and abdominal obesity, which is a risk factor for cardiometabolic disorders independent of BMI, raises concerns about elevated risk for those conditions among resettled Bhutanese in the U.S. These results point to the need for greater focus on screening, diagnosis, treatment, and prevention of excess body weight and abdominal obesity and related chronic diseases among resettled Bhutanese refugees, especially since these conditions will continue to impact their health decades beyond the initial resettlement phase. Moreover, health professionals serving this population may need to be educated about the cardiometabolic risk at a lower BMI for proper counseling of their Bhutanese refugee patients.

The high proportion of excess body weight and abdominal obesity among resettled Bhutanese women is likely due to a combination of pre-immigration factors in the refugee camps in Nepal and post-immigration factors in the U.S. Almost two decades of life in the refugee camps that included a carbohydrate rich diet with rice as a staple food item and a sedentary life style may have contributed to excess body weight [20]. This is a very new population in the U.S. with a median time in the U.S. of about 19 months, and we did not observe a significant association between length of time in the U.S. and either excess body weight or abdominal obesity. This suggests that a certain level of excess weight might have been pre-existing. Unlike other chronic conditions, such as diabetes, which are observed to be associated with longer duration of residency in the U.S. among immigrants [22], excess weight gain may take only a few months to manifest when there is readily available, accessible, and abundant high calorie food and a lack of physical activity. These observations suggest an already existing problem of excess body weight may be exacerbated post-resettlement.

Anecdotal evidence suggests that increased portion sizes, increased frequency of meals, high calorie food intake, unhealthy methods of food preparation (e.g., frying), unbalanced meals, and snacking are common dietary patterns among Bhutanese refugees in the U.S. These observations are consistent with previous studies of diet and physical activity among South Asians, which report diets with high carbohydrates and fat, and low in fruit and vegetable intake, and a lack of physical activity [43,44]. We also observed a strong association (OR = 4.5) of consumption of meat with odds of excess weight. While meat would have been a luxury item consumed very rarely in refugee camps in Nepal, meat is readily available and affordable in the U.S and over 80% of the women reported consuming meat. The additional calories consumed with the addition of meat to a diet already high in carbohydrates and fats may explain the association of meat consumption with excess weight in this group. Moreover, we also observed that a large portion (67%) of women reported consuming sweetened beverages, which is a new dietary adoption in this population and may be contributing to the excess weight. These findings identify a clear need and an opportunity for clinic- and community-based interventions to increase nutrition and physical activity education among Bhutanese refugees during the resettlement process to avert a continued rise in obesity and associated chronic conditions in this population.

The socio-demographic factors significantly associated with excess weight and abdominal obesity included age, marital status, having children, lack of formal education, lack of ability to read English, and being unemployed. Due to the limited sample size, we were unable to assess the independent effect of many of these socio-demographic variables. Since age was highly correlated with marital status, having children, education, and English language skills, we used it as a proxy for the other variables in the multivariable model. Age was significantly positively associated with both total and abdominal obesity with an increase of 0.10 odds per year increase in age. Future research studies with larger sample sizes should assess the independent effect of the other socio-demographic factors to better understand the role of each of these risk factors. From the prevention perspective, however, the observed relationships between the cluster of socio-demographic factors and excess body weight and abdominal obesity in the univariable analyses have implications for designing appropriate clinical and public health intervention programs among women in this group. Older married women with children were more likely to have excess weight but are also likely to have no formal education and ability to read English. Therefore, intervention programs to address the issue of weight among Bhutanese refugee women need to take into account the limited English language skills and low literacy level.

In addition to issues with language and literacy, Bhutanese women may face several additional barriers to adopting behavior change related to diet and physical activity that have been noted among other South Asian immigrant women to western countries. These include gender roles, body image, physical activity misconception, and maintaining cultural identity [45]. Among Bhutanese households, while women are responsible for food preparation, the male and/or older females in the household exert a significant influence on the dietary decisions. Even though a woman may want to change the diet for health reasons, she is unlikely to make modifications that may contradict the family dietary preferences. Similarly, body size with higher BMIs (≥25 kg/m^2^), especially for older and married women in South Asian cultures, are acceptable norms and viewed as a sign of good nutrition and health. Therefore, there is less external pressure to lose weight [46]. Engaging in physical activities for health reasons is not a common phenomenon within most South Asian communities, especially among new immigrants [45]. In addition, because of language, transportation and other cultural and logistical barriers, Bhutanese women are unlikely to engage in physical activities in a traditional setting of a public gymnasium. Moreover, many Bhutanese refugees have been settled in neighborhoods that are not conducive to walking or engaging in similar healthy behaviors due to a lack of public places for walking or safety concerns. Finally, similar to other immigrants, maintaining their dietary practices as a sign of retaining the cultural identity is likely important to Bhutanese as well, especially considering their history of cultural and ethnic persecution. Therefore, modifying dietary practices or cooking methods to make the diet healthier is likely to be challenging and will require innovative intervention approaches that are culturally sensitive and linguistically appropriate. Future research should focus on the extent to which gender role, body image, and maintaining cultural identity influence the issue of excess body weight/abdominal obesity and related prevention efforts for this new immigrant South Asian population in the U.S.

This study has several strengths and limitations and the findings of the study should be interpreted with them in consideration. In terms of the strengths of the study, the questionnaire was developed in the Nepali language and administered face-to-face by trained native Nepali speakers. These methods for instrument development and data collection likely have limited information bias. The study used objective anthropometric measures to assess excess weight and adnominal obesity, thus ensuring limited misclassification of the outcome measures. In terms of the limitations, in addition to being a convenience sample, the sample size was fairly small and limited to women aged 18–65 years, which may limit the generalizability of the study to the entire Bhutanese refugee population. Future studies among adults with a larger sample size that include men and older age groups are recommended. Information on type and frequency of meat and sweetened beverages consumption were not assessed for this study. A single anthropometric measure may have resulted in random measurement errors, thus may have biased the study findings. Therefore, future studies with more specific measures of dietary practices and multiple anthropometric measures are recommended to gain a better understanding of dietary practices and minimize errors.

### Conclusions

The high prevalence of excess body weight and abdominal obesity, even at this early stage of resettlement among the Bhutanese refugee women in the U.S., is a matter of concern for the short- and long-term health of this population. As the population acculturates to the lifestyle in the U.S., obesity is likely to increase and associated chronic diseases are also likely to have greater impact on the health of this new immigrant group. This phenomenon is well documented among other South Asian immigrants to western countries, who are shown to have higher rates of diabetes and cardiovascular diseases than other ethnic groups [22,23,24,25,26,27]. This highlights the need for public health intervention programs among this new immigrant population to reduce the health consequences of excess body weight and abdominal obesity. There are clearly challenges related to socio-economic and cultural barriers in designing appropriate clinical and/or community-based dietary and life-style change interventions. Understanding the constraints and barriers will assist in developing culturally appropriate behavior change programs to deal with the issue of obesity and its health consequences in this new, vulnerable, and health disparate immigrant group to the U.S.
